# The direct and urinary electrolyte-mediated effects of ambient temperature on population blood pressure: A causal mediation analysis

**DOI:** 10.1016/j.envint.2024.109208

**Published:** 2025-01

**Authors:** Ayesha Mukhopadhyay, Momenul Haque Mondol, Mahbubur Rahman, Leanne Unicomb, Rizwana Khan, Hoimonty Mazumder, Mohammad Nahian Ferdous, Emily V. Pickering, Konstantinos C. Makris, Alberto J. Caban-Martinez, Faruk Ahmed, Mohammad Shamsudduha, Fawaz Mzayek, Chunrong Jia, Hongmei Zhang, Anwar Musah, Lora E. Fleming, Matthew P. Smeltzer, Howard H. Chang, John L. Jefferies, Csaba P. Kovesdy, Xichen Mou, Abu Mohd Naser

**Affiliations:** a Division of Epidemiology, Biostatistics, and Environmental Health, School of Public Health, The University of Memphis, Memphis, TN, USA; b School of Population and Public Health, University of British Columbia, Vancouver, BC Canada; c Department of Statistics, University of Barishal, Barishal, Bangladesh; d International Centre for Diarrheal Disease Research, Bangladesh (icddr,b), Dhaka 1212, Bangladesh; e Cyprus International Institute for Environmental and Public Health, School of Health Sciences, Cyprus University of Technology, Limassol, Cyprus; f Department of Public Health Sciences, University of Miami, Miller School of Medicine, Miami, FL, USA; g Department of Engineering Technology, The University of Memphis, Memphis, TN, USA; h Department of Risk and Disaster Reduction, University College London, London, UK; i Department of Geography, University College London, London, UK; j European Centre for Environment and Human Health, University of Exeter Medical School, Penryn, Cornwall, United Kingdom; k Department of Biostatistics & Bioinformatics, and Gangarosa Department of Environmental Health, Rollins School of Public Health, Emory University, Atlanta, GA, USA; l Division of Nephrology, University of Tennessee Health Science Centre, Memphis, TN, USA

**Keywords:** Climate Change, Global Warming, Environmental Health, Ambient Temperature, Blood Pressure, Nephrology, Cardiorenal Physiology, Urinary Electrolytes

## Abstract

High ambient heat can directly influence blood pressure (BP) through the vasodilation of the skin vasculature and indirectly by affecting urinary volume and electrolyte levels. We evaluated the direct and urine electrolyte-mediated effects of ambient temperature on BP. We pooled 5,624 person-visit data from a community-based stepped-wedge randomized control trial in southwest coastal Bangladesh from December 2016 to May 2017. Same-day ambient temperature data from local weather stations were linked to participant BP and urine electrolytes using geo-locations of their residential addresses. We implemented causal mediation analyses using the product methods of coefficients with linear mixed models under the sequential ignorability assumption. Separate models were run for each urinary electrolyte mediator (sodium, potassium, calcium, and magnesium), followed by combined models to evaluate the natural direct and electrolyte-mediated indirect effects of temperature on BP. Models had participant-level random intercepts and were adjusted for age, sex, body mass index (BMI), religion, exercise, smoking status, sleep hours, alcohol consumption, urine creatinine, time trend, household assets, drinking water salinity, and seasonality. For the combined mediators (sodium, potassium, calcium, and magnesium), for every 5°C increase in average daily temperature: the direct effect on systolic BP was −1.42 (95 % CI: −1.94, −0.92) mmHg and urine sodium mediated effect was −0.12 (95 % CI: −0.20, −0.05) mmHg; while urine potassium mediated effect was 0.15 (95 % CI: 0.08, 0.25) mmHg; urine calcium-mediated effect 0.06 (95 % CI: 0.01, 0.12) mmHg; and urine magnesium mediated effect −0.00 (95 % CI: −0.03, 0.02) mmHg. We detected similar associations for diastolic BP, pulse pressure, and mean arterial pressure. We found a significant inverse direct effect of ambient temperature on BP compared to minimally mediated urine electrolyte effects. Further studies are needed to uncover the underlying mechanisms of ambient heat and BP associations and to describe the clinical consequences of these associations.

## Highlights

• Ambient temperature exposure inversely affects the population's blood pressure.
• The urine electrolyte-mediated indirect effect of ambient temperatures on all BP was negligible.
• Excessive temperature in residences and workplaces can substantially lower individuals' blood pressure.

## 1 Introduction

Elevated blood pressure (BP) is the leading cause of disability and premature death globally. (Lewington et al., 2012, Gostimirovic et al., 2020) According to The Global Burden of Diseases, Injuries, and Risk Factors (GBD) Study, age-standardized disability adjusted-life years (DALYs) due to elevated BP was 2,770 per 100,000 (95 % confidence interval: 2,310–3,160 per 100,000) in 2021. (Vaduganathan et al., 2022) A small rise in the mean population BP can translate into a substantial public health burden of cardiovascular diseases (CVDs), and therefore, a modest pro-hypertensive role of pervasive environmental factors can have a major impact on the CVD burden. Many communities worldwide are increasingly experiencing extreme temperatures because of global climate change. July 2023 was recorded as the world’s hottest month ever, representing the transition to an “era of global boiling”, as per the United Nations Secretary-General. (United Nations, 2023) Variations in ambient temperature due to climate change can directly influence population-level BP through a range of mechanisms. (Gostimirovic et al., 2020, Wang et al., 2017, Goyal et al., 2019, Ganio et al., 2011, Chen et al., 2013).

Epidemiological studies report a higher prevalence of cardiovascular morbidity and mortality due to increased BP during the winter. (Wang et al., 2017, Goyal et al., 2019, Chen et al., 2013, Brennan et al., 1982, Sharma et al., 1990, Sega et al., 1998, Radke and Izzo, 2010, Nayha, 1985) Several large-scale studies demonstrate a strong inverse association between ambient temperature and population mean BP, (Lewington et al., 2012, Brennan et al., 1982, Sharma et al., 1990, Sega et al., 1998, Radke and Izzo, 2010, Nayha, 1985, Park et al., 2020, Radin et al., 2018) indicating a lower mean population BP when the ambient temperature is higher. Putative physiological mechanisms for lower BP during the summer compared to winter include temperature-induced skin vasodilatation as part of physiological thermoregulation, causing reduced total peripheral resistance and decreased BP. (Johnson, 1996, Chen et al., 2013) With significant increases in cardiac output (up to 10 L/min or more), the blood flow is directed from the central circulation (e.g., splanchnic and renal flow) to the periphery and skin. (Kihara, 2007) Skin blood flow can increase from 5 % to 60 % of cardiac output in response to heat. (Johnson, 1996).

High ambient temperature also influences sweat production, and water and electrolytes loss through sweating. (Johnson, 1996, Chen et al., 2013) A net negative water or electrolyte balance during seasons of high ambient heat due to sweating results in lower extracellular volume, however, compensatory renal mechanisms influence urinary water or electrolyte reabsorption. Urinary volume and electrolyte excretion due to ambient temperature exposure reflect the kidney response to non-renal water and electrolyte losses, and can thus are reflective of systemic fluid and electrolyte balance. (Abuelo, 2007) Epidemiological studies suggest that electrolyte concentrations in urine are associated with population BP. (Jackson et al., 2018, Naser et al., 2020).

Currently, a scientific knowledge gap exists about the significance of the direct effect of ambient temperature on BP (e.g., vasodilatation effect) and indirect electrolyte-mediated influence of ambient temperature on BP (e.g., effect on water and electrolyte balance) (Supplemental Fig. 1). A scientific evaluation of the direct effect and electrolyte-mediated effect of ambient temperature on observed BP changes is important for understanding the most significant pathways of BP regulation in response to ambient temperature change and for developing climate adaptive interventions. In addition, we also lack evidence about which electrolyte (e.g., sodium, potassium, calcium, and magnesium) has the most significant role in BP regulation when ambient temperature changes. The overarching aim of this study is to assess and compare the magnitude of direct and electrolyte (sodium, potassium, calcium, and magnesium) mediated effects of ambient temperature on systolic and diastolic BP, pulse pressure, and mean arterial pressure using causal mediation analyses in tropical southwest coastal Bangladesh.

## 2 Methods

***Study population.*** We used data from a stepped-wedge cluster randomized trial in southwest Bangladesh's three tropical coastal districts (Mongla, Khulna, and Satkhira). (Naser et al., 2017, Naser et al., 2021) 1,175 participants of ≥ 20 years of age from 542 households and 16 communities were followed for five monthly visits from December 2016 to April 2017, which covered winter and early summer in Bangladesh. In total, 5,624 person-visit data from five visits were included in the analysis. Details of the study design and participation are reported elsewhere. (Naser et al., 2017) The first visit was conducted between November 17, 2016, and December 20, 2016; the second visit was between December 21, 2016, and January 26, 2017; the third visit was between February 02, 2017, and March 02, 2017; the fourth visit between March 03, 2017, and April 04, 2017; and the fifth visit between April 05, 2017, and May 05, 2017. (Naser et al., 2020).

***Exposure data.*** Southwest coastal Bangladesh is 1.5–11.8 m above sea level. January is the coldest month in the region, and June is the warmest month. (Tagliamento et al., 2021, Dai et al., 2020) Data on the daily ambient temperature of the communities were collected from the Bangladesh Meteorological Board (BMD). (Bangladesh Meterological Department) During the study period, we collected data for daily average, maximum, and minimum temperatures from the three regional weather stations in the study areas (Mongla, Khulna, Satkhira). These data were spatiotemporally linked based on the participant residencies and proximity to local weather stations. We considered each participant’s residential address and calculated three linear distances from the three local weather stations. Then, daily temperature data were assigned to each participant using the weather station with the shortest linear distance from their residence. Since the influence of temperature on BP and urine electrolytes occurs within hours, we considered the same-day temperature as when the BP was measured as the exposure.

***Blood pressure data.*** At every visit, participants’ systolic blood pressure (SBP) and diastolic blood pressure (DBP) were measured using an Omron HEM-907 monitor. Pulse pressure (PP), defined as the difference between systolic and diastolic blood pressures, and mean arterial pressure (MAP), defined as diastolic blood pressure + 1/3 pulse pressure, were also calculated.

Participants were asked to refrain from drinking caffeinated beverages, smoking, vigorous physical activity, and eating, half an hour before BP measurement. BP was measured between 7:30 am-2:00 pm after 5-minute resting in the sitting position and recorded as the mean of three consecutive measurements. (Naser et al., 2019).

***Urine electrolyte data.*** We used daily urine electrolyte excretion (e.g., 24-hour sodium, potassium, calcium, and magnesium excretions) as the mediators since they are a good proxy of water and electrolyte balance. We considered 24-hour electrolytes as a better proxy mediator of electrolyte balance than spot-urine electrolyte excretions since hypohydration in response to ambient heat exposure can increase urine concentrations of electrolytes due to urinary water reabsorption. However, urine volume is factored in 24-hour urine electrolytes, minimizing the bias due to hypohydration.

During each visit, we collected 24-hour urine to measure electrolytes (sodium, potassium, calcium, and magnesium). (Naser et al., 2020) Each participant was given a 4-liter plastic container and a mug to transfer the 24-hour voided urine into the plastic container. (Naser et al., 2020) They were directed to discard the first-morning void and start collection from the second void. Field research assistants documented each participant’s 24-hour urine volume, and 15 ml of urine was obtained from the 4-liter container after adequate stirring. The urine samples were transported for processing and analysis to field laboratories on the same day at 2–8 °C.

Direct ion selective electrode methods using a semi-auto electrolyte analyzer (Biolyte 2000, Bio-care Corporation, Taiwan, coefficient of variation: ±5%) were used for measuring urinary sodium and potassium concentrations. For calcium and magnesium measurements, we used photometric titration methods with a semi-auto biochemistry analyzer (Evolution 3000, BSI, Italy, coefficient of variation: <1%). We obtained 24-hour urine electrolyte excretion by multiplying the participant's 24-hour urine volume with concentrations.

***Covariates data.*** We collected participants’ data on age, gender (male vs. female), religion (Muslim vs. Hindu), and household asset data. Participants self-reported tobacco use (no, current, and former), physical activity levels using the WHO Global Physical Activity Questionnaire (vigorous, moderate, and sedentary), average hours of sleep in 24 h (less than 6 h, 6 h to 9 h, and greater than or equal to 9 h), and alcohol consumption (yes vs. no) data were also collected during each visit. We measured 24-hour urine creatinine excretion and stored drinking water salinity (temperature-adjusted electrical conductivity at 25°C with a Hanna Salinity meter) data during each visit. We measured participant weight in all visits using a Seca weight machine (accuracy: 0.05–0.1 kg; model: 874–1321009, Hamburg, Germany) and height in the first visit with Shorr board (accuracy: 1/8″ or 0.1 cm; Olney, Maryland). BMI was calculated as weight (kg)/height (m^2^). We also collected the daily humidity data from the Bangladesh Meteorological Board.

**Fig. 1:**
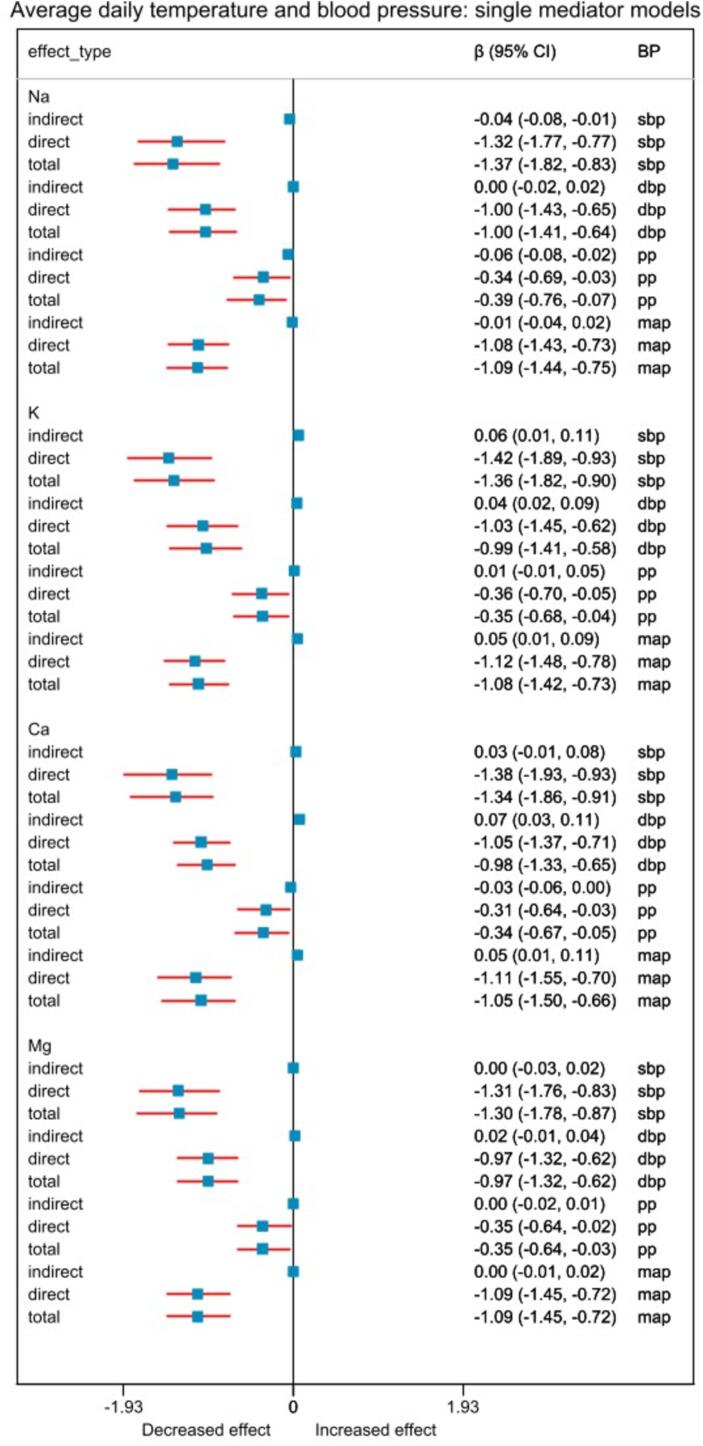
Relationship between 5 °C increase in average daily temperature and blood pressure when single mediator models were used. “direct” effect indicates natural direct effect, and “indirect” indicates natural indirect effect. Abbreviations: Na, Sodium; K, potassium; Ca, Calcium; Mg, Magnesium; β, effect estimate; 95 % CI, 95 % confidence interval; BP, blood pressure; sbp, systolic blood pressure; dbp, diastolic blood pressure; pp, pulse pressure; map, mean arterial pressure.

***Statistical analyses.*** We calculated the household wealth index based on self-reported information on ownership of motorbikes, bicycles, cell phones, refrigerators, televisions, air conditioners, chairs, tables, sewing machines, motor pumps, wristwatches, wooden cots, motorized rickshaws, rice husking machines, and access to electricity. We used principal component analysis to derive household asset scores and then categorized them into quintiles. (Ben Salem and Ben, 2021) We used separate boxplots to visualize the average daily ambient temperature and BP across visits (Figs. 1a and b). The Pearson correlation test was used to determine the correlation between the daily temperatures of the three weather stations.

Using the same data, we elsewhere demonstrated that ambient temperature influences the excretion of 24-hour urine electrolytes, (Mazumder et al., 2024) justifying the use of urinary electrolytes as mediators in our analyses in this paper. For each potential mediator (urinary sodium, potassium, calcium, and magnesium), we evaluated the natural direct and natural indirect effects of ambient temperature on BP by implementing causal mediation analyses considering the sequential ignorability assumption, (Rudolph et al., 2019) but did not include interaction terms between ambient temperature and urine electrolytes.

Under the counterfactual framework, the causal effect of exposure on the outcome is the hypothetical difference between the health outcomes observed in an individual at the same time at two different magnitudes of exposure. (Richiardi et al., 2013) If Ya is the outcome under the exposure “a” and Ya* is the outcome under the exposure “a*” (a ≠ a*), the individual causal effect is (Ya − Ya*). Nevertheless, Ya and Ya* outcomes occur under different exposure levels; hence, it is impossible to observe both Ya and Ya* in the same individual simultaneously (only one of the two outcomes would be factual). For mediation analysis, if Ya,m is the outcome for exposure “a” and the mediator is “m”, the natural direct effect (NDE) is the difference between the counterfactual health outcomes observed in an individual when exposure is “a” versus exposure is “a*”, and value of mediator set at the reference exposure “a*” [Ya,m(a*)-Ya*, m(a*)]. (Richiardi et al., 2013) The natural indirect effect (NIE) is the difference between the counterfactual health outcomes observed in an individual when exposure is “a”, and the value of mediator set at the reference exposure “a” versus the value of mediator set at the reference exposure “a*” [Ya,m(a)-Ya, m(a*)]. (Richiardi et al., 2013).

The *mediation* R package was used that relied on the Product Method of coefficients proposed by Baron and Kenny. (Tingley et al., 2014, Baron and Kenny, 1986) We used linear mixed models, with participant-level random intercepts, for coefficients; estimation, and quasi-Bayesian Monte Carlo simulation on normal approximation for the confidence intervals. (Imai et al., 2010) Although a nonlinear relationship exists between temperature and mortality, the relationship between temperature and BP is somehow linear (Halonen et al., 2011) based on temperature’s impact on vascular response. Hence, we only considered the linear exposure-outcome relationship in our analyses. The sequential ignorability assumption implies that there is no unmeasured confounding of the treatment-mediator, treatment-outcome, and mediator-outcome relationships; and the mediator-outcome relationship is unaffected by the change in exposure. (Rothman et al., 2021) To fulfill the sequential ignorability assumption, we adjusted the statistical models for a comprehensive list of potential confounders, which included age, sex, BMI, religion, physical activity, tobacco use, hours of sleep, alcohol use, household wealth, drinking water salinity, urine creatinine levels, humidity, and time trend and seasonality. Although average daily temperature is the primary exposure, we also ran models for the maximum and minimum daily temperatures. We initially implemented separate models for single electrolyte mediators and then included all electrolyte mediators in the same model.

We conducted sensitivity analyses for the possible existence of unobserved confounders for which the sequential ignorability assumption would be violated using the *medsens* function in the R *mediation* package. We first selected the sensitivity parameter (ρ) (the correlation between the residuals of the mediator and outcome regressions) by implementing linear regression. If unobserved confounders were present, the sequential ignorability assumption will be violated and “ρ” will deviate from zero. Consequently, we examined the observed direct and direct effects at different values of “ρ”.

We performed a second sensitivity analysis after excluding participants who reported using antihypertensive medications. Since our outcome is blood pressure, our results could be biased if study participants were antihypertensive medication users. Moreover, some antihypertensive medications influence urine electrolyte concentrations. For example, thiazide and loop diuretics increase sodium and potassium excretion in urine. (Spital, 1999, Friedman et al., 1989, Sonnenblick et al., 1993, Clark et al., 1994) Hypertensive medications are also used in other diseases conditions such as renal insufficiencies, cardiovascular diseases, and stroke. Hence, we conducted stratified analyses using the multiple mediators of 24-hour urine excretions after excluding participants who used antihypertensive medications. Thirdly, we performed a third sensitivity analysis including only participants who had complete 24-hour urine samples based on creatinine index > 0.7, to account for over-, or under-collection of the 24-hour urine samples. (Naser et al., 2020, John et al., 2016).

Finaly, to provide a broader perspective of the relationship between ambient temperature, urine electrolytes and blood pressure, we added sensitivity analyses by including interaction terms between the exposure and mediator in the regression model that may indirectly provide insights into the mediation. Our interpretation was that if the interaction term was statistically significant, this may indicate a pathway through the mediator. However, we understood that this approach lacks the formal causal interpretation of causal mediation analysis. Under such sensitivity analyses, we implemented linear mixed model with participant level random intercept (restricted maximum likelihood estimation) and used interaction term between ambient temperature and urine electrolyte (24-hour sodium and potassium) to evaluate if the association between ambient temperature and blood pressure (systolic and diastolic blood pressure) is modified by different values of 24-hour urine sodium and potassium.

***Ethical considerations.*** The Ethical Review Committee (ERC) of the International Center for Diarrheal Disease Research, Bangladesh reviewed and approved the protocol (PR # 15096). Household heads and individual participants gave written informed consent for study participation. To ensure the maintenance of participant confidentiality, we used de-identified the data for our analyses.

## 3 Results

***Participants.*** The mean age and BMI of the participants were 42.9 years (95 % CI: 42.6, 43.3) and 22.2 kg/m^2^ (95 % CI: 22.06, 22.26) (Table 1). The majority (59.4 %) of the participants were women; 58.1 % were Hindus; 40 % reported rigorous physical activity; 51 % did not use tobacco; 67 % slept more than six hours/day; and 97 % did not consume alcohol. Other characteristics of the participants are listed in Table 1. None of the participants’ households had air conditioning.

**Table 1:** Participants characteristics and urine biomarkers during the baseline visit.

**Characteristics**	**Overall (N = 1175)**
Age (Mean, 95 % CI), years	42.9 (42.6–43.3)
BMI (Mean, 95 % CI), kg/m2	22.2 (22.06–22.26)
Female participants	59.4 (698)
Religion (%, n)	
Islam	41.9 (492)
Hindu	58.1 (683)
Smoking (%, n)	
Never	50.7 (596)
Former	9.1 (107)
Current	40.2 (472)
Alcohole use (%, n)	
Yes	2.89 (34)
No	97.1 (1141)
Physical exercise (%, n)	
Sedentary	40.4 (475)
Moderate	31.2 (366)
Vigorous	28.4 (334)
Sleep (%, n)	
<6 h	21.1 (248)
6 to > 9 h	66.8 (785)
≥ 9 h	12.1 (142)
Household wealth quintile (%, n)	
1st quintile	17.6 (207)
2nd quintile	18.4 (216)
3rd quintile	19.2 (225)
4th quintile	20.8 (244)
5th quintile	24.1 (283)
Urinary creatinine concentration in mmol/L (Median, IQR)	10.9 (8.3–14.0)
24-hour urinary creatinine excretion in mmol/L, (Median, IQR)	21.2 (12.9–33.4)
24-hour urinary excretion of urine biomarkers (Median, IQR)	
Sodium (mmol/24 h)	157.8 (118.6–207.4)
Potassium (mmol/24 h)	32.5 (24.0–42.2)
Chloride (mmol/24 h)	166.9 (126.8–222.4)
Calcium (mmol/24 h)	3.4 (1.9–5.4)
Magnesium (mmol/24 h)	3.3 (2.1–4.8)
Urine total protein (mg/24 h)	207.7 (122.4–326.2)
Urine volume (liter/24 h)	1.9 (1.4–2.6)

***Ambient temperature and blood pressure.*** The mean (and range) of average ambient temperature during the first, second, third, fourth, and fifth visits were: 21.1 °C (18.6 – 23.6), 19.4 °C (16.6 – 21.7), 23.3 °C (19.8 – 27.4), 26.0 °C (21.6 – 30.4), and 29.0 °C (26.1 – 32), respectively (Supplemental Fig. 2a). The pair-wise Pearson correlation coefficients between the average daily temperature of weather stations were > 0.99 (Supplemental Fig. 3).

The mean systolic and diastolic BP of the participants during the first visit (Nov-Dec 2016) were 115.6 mmHg (95 % CI: 114.6 to 116.6) and 68.9 mmHg (95 % CI: 68.3 to 69.6). The mean systolic and diastolic BP of the participants during the fifth visit (Apr-May 2017) were 110.2 mmHg (95 % CI: 109.3 to 111.1) and 65.8 mmHg (95 % CI: 65.3 to 66.5) (Supplemental Fig. 2b).

***Direct and urine electrolyte mediated effects of ambient temperature on blood pressure.*** In the adjusted models for each 5 °C increase in average daily temperature, the natural direct and urine sodium-mediated indirect effects of average daily temperature on systolic BP were: −1.32 (95 % CI:-1.77, −0.77) mmHg and −0.04 (95 % CI: −0.08, −0.01) mmHg; natural direct and potassium-mediated indirect effects were −1.42 (95 % CI: −1.89, −0.93) mmHg and 0.06 (95 % CI: 0.01, 0.11) mmHg; natural direct and calcium-mediated indirect effects were −1.38 (95 % CI: −1.93, −0.93) mmHg and 0.03 (95 % CI: −0.01, 0.08) mmHg, and natural direct and magnesium-mediated indirect effects were −1.31 (95 % CI: −1.75, −0.83) mmHg and −0.004 (95 % CI: −0.03, 0.02) mmHg (Fig. 1).

For each 5 °C increase in average daily temperature, the natural direct and urine sodium-mediated indirect effects of average daily temperature on diastolic BP were: −1.00 (95 % CI: −1.43, −0.65) mmHg and 0.00 (95 % CI: −0.02, 0.02) mmHg; natural direct and potassium-mediated indirect effects −1.03 (95 % CI: −1.45, −0.62) mmHg and 0.04 (95 % CI: 0.02, 0.09) mmHg; natural direct and calcium-mediated indirect effects −1.05 (95 % CI: −1.37, −0.71) mmHg and 0.07 (95 % CI: 0.03, 0.11) mmHg; and natural direct and magnesium-mediated indirect effects −0.97 (95 % CI: −1.32, −0.62) mmHg and 0.04 (95 % CI: −0.01, 0.02) mmHg in full adjusted models (Fig. 1).

With each 5 °C increase in average daily temperature, the natural direct and urine sodium-mediated indirect effects of average daily temperature on pulse pressure were: −0.34 (95 % CI: −0.69, −0.03) mmHg and −0.06 (95 % CI: −0.08, −0.02) mmHg; natural direct and potassium-mediated indirect effects −0.36 (95 % CI: −0.70, −0.05) mmHg and 0.01 (95 % CI: −0.01, 0.05) mmHg; natural direct and calcium-mediated indirect effects −0.31 (95 % CI: −0.64, −0.03) mmHg and −0.03 (95 % CI: −0.06, 0.00) mmHg; and natural direct and magnesium-mediated indirect effects −0.35 (95 % CI: −0.64, −0.02) mmHg and −0.00 (95 % CI: −0.02, 0.01) mmHg in fully adjusted models (Fig. 1).

**Fig. 2:**
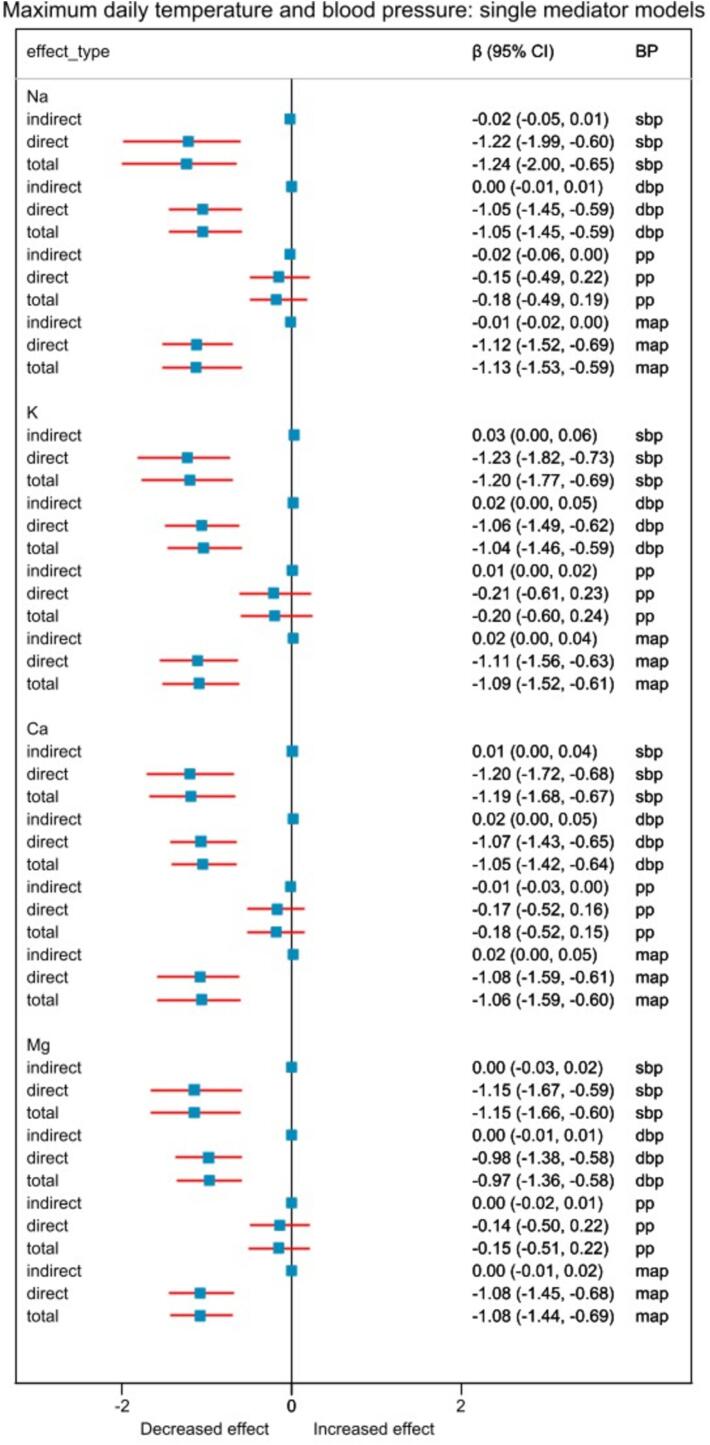
Relationship between 5 °C increase in maximum temperature and blood pressure when single mediator models were used. “direct” effect indicates natural direct effect, and “indirect” indicates natural indirect effect. Abbreviations: Na, Sodium; K, Potassium; Ca, Calcium; Mg Magnesium; β, effect estimate; 95 % CI, 95 % confidence interval; BP, blood pressure; sbp, systolic blood pressure; dbp, diastolic blood pressure; pp, pulse pressure; map, mean arterial pressure.

**Fig. 3:**
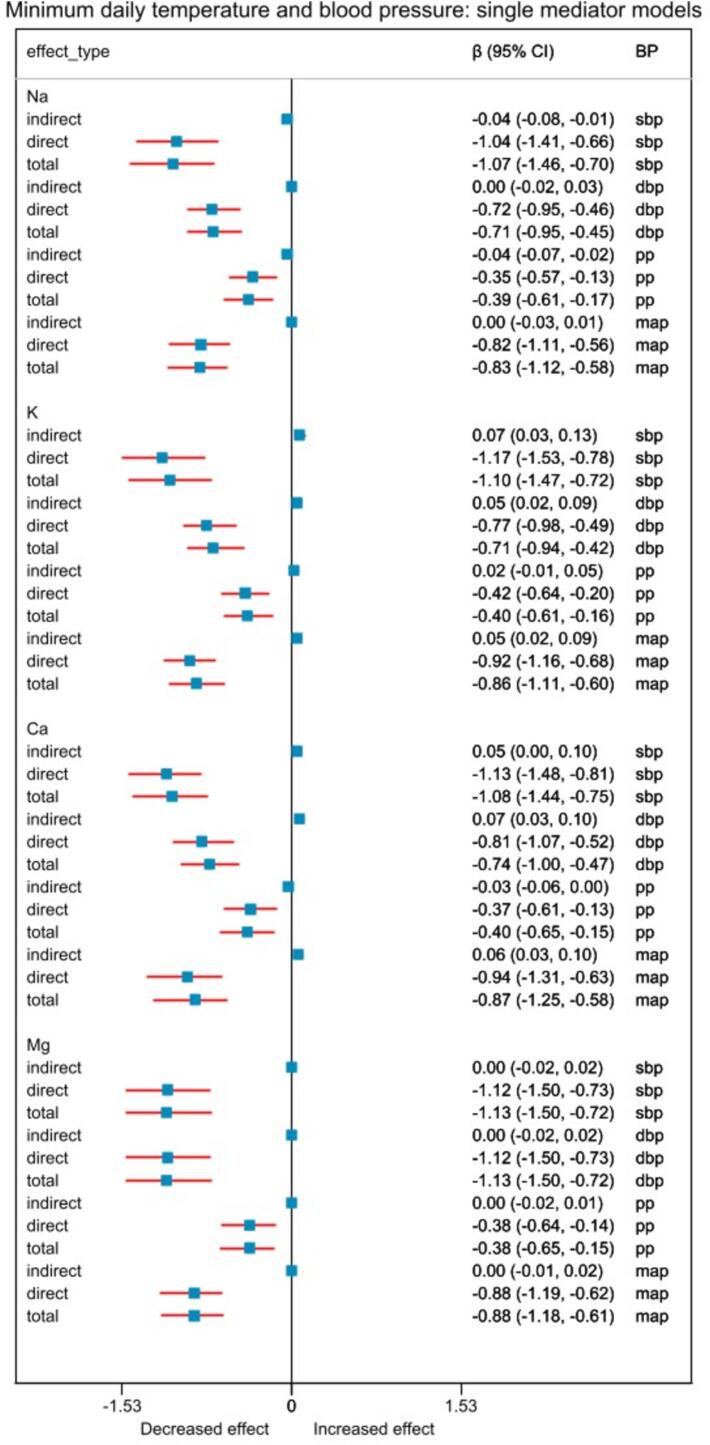
Relationship between 5 °C increase in minimum temperature and blood pressure when single mediator models were used. “direct” effect indicates natural direct effect, and “indirect” indicates natural indirect effect. Abbreviations: Na, Sodium; K, Potassium; Ca, Calcium; Mg, Magnesium; β, effect estimate; 95 % CI, 95 % confidence interval; BP, blood pressure; sbp, systolic blood pressure; dbp, diastolic blood pressure; pp, pulse pressure; map, mean arterial pressure.

From the full adjusted model, for every 5 °C increase in average daily temperature, the natural direct and urine sodium-mediated indirect effects of average daily temperature on mean arterial pressure were: −1.08 (95 % CI: −1.43, −0.73) mmHg and −0.01 (95 % CI: −0.04, 0.02) mmHg; natural direct and potassium-mediated indirect effects −1.12 (95 % CI: −1.48, −0.78) mmHg and 0.05 (95 % CI: 0.01, 0.09) mmHg; natural direct and calcium-mediated indirect effects −1.11 (95 % CI: −1.55, −0.70) mmHg and 0.05 (95 % CI: 0.01, 0.11) mmHg; and natural direct and magnesium-mediated indirect effects −1.09 (95 % CI: −1.45, −0.72) mmHg and −0.00 (95 % CI: −0.01, 0.02) mmHg in fully adjusted models (Fig. 1). We had similar findings for maximum (Fig. 2) and minimum temperatures (Fig. 3).

**Fig. 4:**
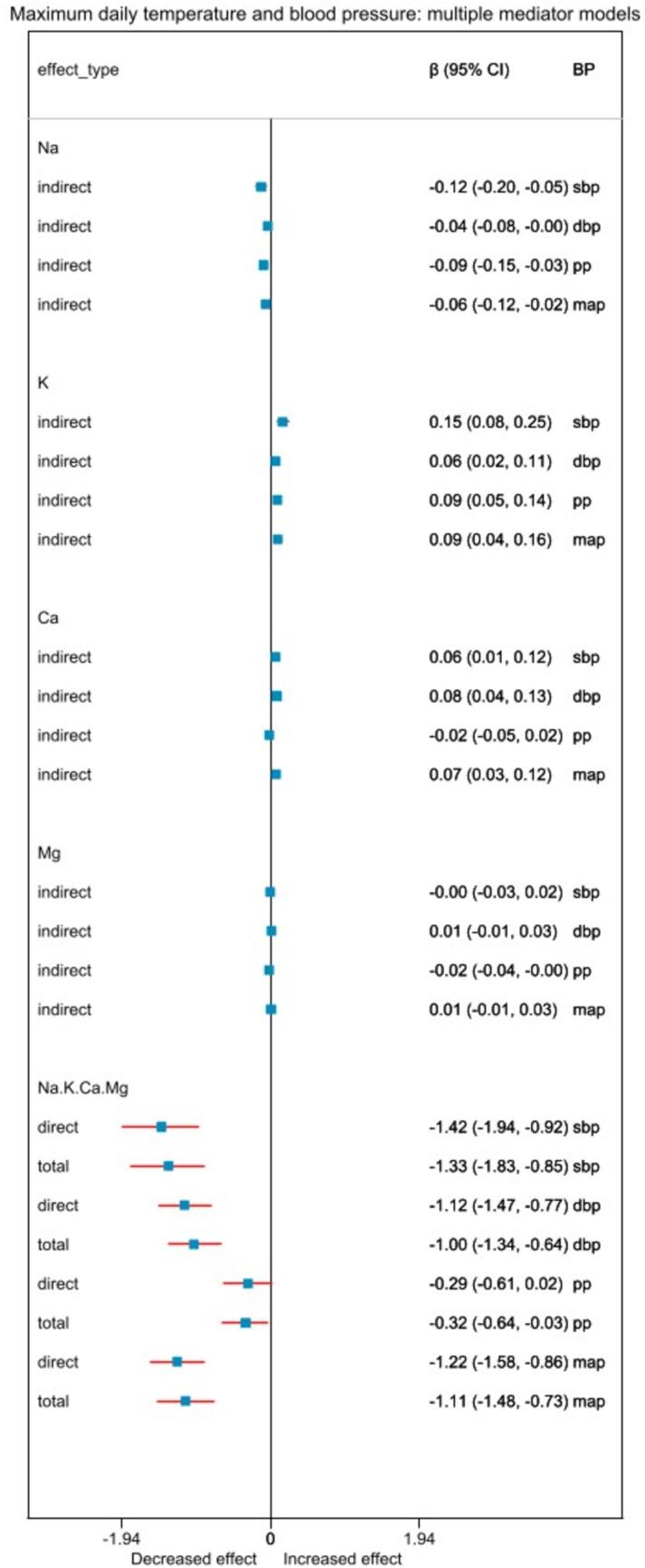
Relationship between 5 °C increase in average daily temperature and blood pressure when multiple mediators were used in the same model. “direct” effect indicates natural direct effect, and “indirect” indicates natural indirect effect. Abbreviations: Na, Sodium; K, Potassium; Ca, Calcium; Mg, Magnesium; β, effect estimate; 95 % CI, 95 % confidence interval; BP, blood pressure; sbp, systolic blood pressure; dbp, diastolic blood pressure; pp, pulse pressure; map, mean arterial pressure.

When all mediators were used in the full adjusted model, for every 5 °C increase in average daily temperature, the natural direct effect on systolic BP was: −1.42 (95 % CI: −1.94, −0.92) mmHg; urine sodium-mediated indirect effect −0.12 (95 % CI: −0.20, −0.05) mmHg; urine potassium-mediated indirect effect 0.15 (95 % CI: 0.08, 0.25) mmHg; urine calcium-mediated indirect effect 0.06 (95 % CI: 0.01, 0.12) mmHg; and urine magnesium-mediated indirect effect −0.00 (95 % CI: −0.03, 0.02) mmHg (Fig. 4). Similarly, for every 5 °C increase in average daily temperature, the natural direct effect on diastolic BP was: −1.33 (95 % CI: −1.83, −0.85) mmHg; urine sodium-mediated indirect effect −0.04 (95 % CI: −0.08, −0.00) mmHg; urine potassium-mediated indirect effect 0.06 (95 % CI: 0.02, 0.11) mmHg; urine calcium-mediated indirect effect 0.08 (95 % CI: 0.04, 0.13) mmHg; and urine magnesium-mediated indirect effect 0.01 (95 % CI: −0.01, 0.03) mmHg.

**Fig. 5:**
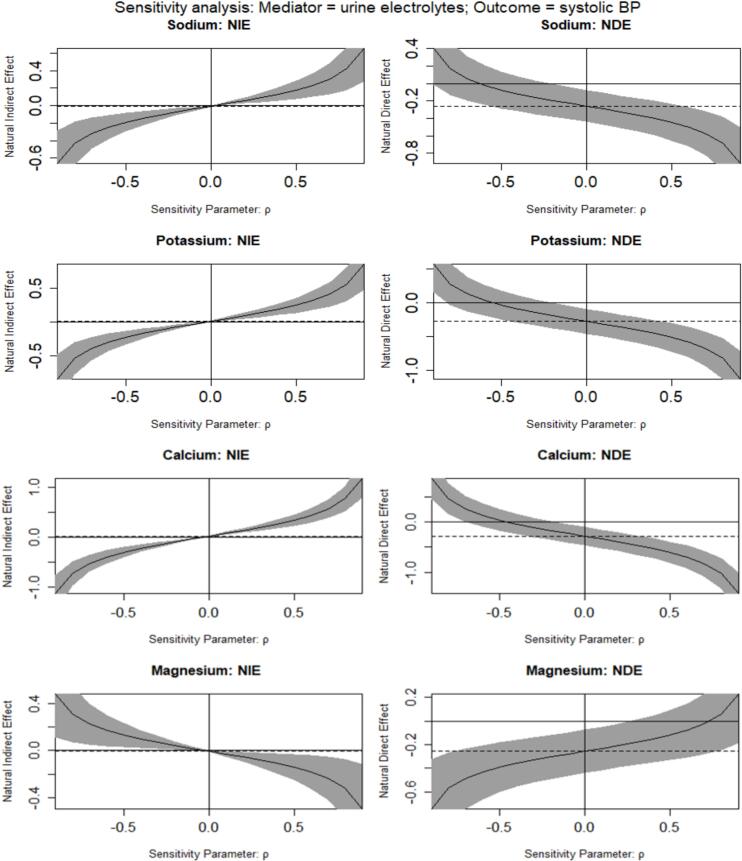
Sensitivity analyses for the presence of unobserved confounding between mediator and outcome. The sensitivity parameter “ρ” is the correlation between the residuals of the mediator and outcome regressions used in causal mediation analyses to identify two coefficients due to a 1 °C increase in ambient daily temperature. NIE means natural indirect effect and NDE means natural direct effect. The dotted lines represent the value of NIE and NDE under the sequential ignorability assumption. The solid lines and 95 % confidence intervals (greyish areas) are the values of NIE and NDE at different “ρ”.

Sensitivity analyses suggest that the natural urine electrolyte-mediated indirect effect of average daily ambient temperature on systolic BP is highly sensitive to the presence of unmeasured confounders influencing the studied associations. If the sensitivity parameter ρ is different from zero, the natural mediated indirect effect varies for each of the urine electrolyte mediators (Fig. 5). For example, the urine sodium-mediated effect of average daily ambient temperature on systolic BP becomes positive if “ρ” is positive and becomes negative if “ρ” is negative. Nevertheless, the natural direct effect of ambient temperature on systolic BP is less sensitive to the magnitude of “ρ”. The natural direct effects of ambient temperature on systolic BP remain negative unless the value of “ρ” is less than 0.6.

We found similar findings of the direct and electrolyte-mediated effect of ambient temperature when we implemented stratified analyses after excluding study participants who reported the use of anti-hypertensive medication (Supplemental Table 1) or when restricted the analyses among participants with complete 24-hour urine sample collection (Supplemental Table 2). We did not find any interaction term for the exposure and mediator statistically significant (e.g., p-value for Wald test > 0.05) for the relationship between daily temperature and systolic and diastolic blood pressure (Supplemental Table 3).

## 4 Discussion

Our results demonstrate that the natural direct effect of daily ambient temperature on all types of BP is higher and statistically significant compared to the natural urine electrolytes (sodium, potassium, calcium, and magnesium) mediated indirect effect of temperature on BP. We observed a strong negative association with ambient temperature and the four BP parameters. The urine electrolyte-mediated indirect effect of all ambient temperatures on BP was negligible and did not differ across the four (sodium, potassium, calcium, and magnesium) mediators evaluated.

Our findings were consistent with other studies that evaluated the direct effect of ambient temperature on BP. A study in China that looked at five urban and five rural areas found outdoor temperature to be inversely and strongly associated with systolic BP (for every 10 °C decrease in ambient temperature, systolic BP increased by 5.7 [standard error 0.04] mmHg). (Lewington et al., 2012) This study also found the mean difference of systolic BP between summer and winter months to be 10 mmHg and was more pronounced in rural areas. A longitudinal study that followed hypertensive patients for three years found that ambient temperature was inversely associated with both systolic BP [-0.26 (95 % CI: −0.35, −0.18) mmHg] and diastolic BP [-0.17 (95 % CI: −0.22, −0.12) mmHg], with the average temperature fluctuations between 2.3–31.2 °C. (Chen et al., 2013) A clinic-based study conducted in Italy similarly found one degree Celsius increase in ambient temperature was associated with − 0.14 (95 % CI: −0.25, −0.02) mmHg change in daytime systolic BP.

Another study performed in non-institutionalized elderly individuals across three French cities found that systolic BP decreased between the lowest and highest temperature quintiles (for every 15 °C decrease in ambient temperature, systolic BP increased by 0.8 mmHg). (Alperovitch et al., 2009) A population-based cross-sectional study that looked at the effects of seasonality on BP found that elderly individuals who lived in rural parts of Northern India had significantly increased systolic (p < 0.001) and diastolic (p < 0.001) BP when ambient temperature was low. This study also found a higher prevalence of hypertension during the winter months when compared to summer. (Goyal et al., 2019).

A *meta*-analysis that assessed the effect of ambient temperature on BP found statistically significant and inverse associations between ambient (average, maximum, and minimum) temperatures and BP (systolic and diastolic) and more pronounced effects among those with concomitant cardiovascular comorbidities.^5^ The direct effect of ambient temperature on BP can be explained by several mechanisms of thermoregulation. First, excessive ambient temperature triggers thermoreceptors that cause vasodilation so that blood flow is enhanced toward the skin, enabling excess body heat to escape. Vasodilation decreases systemic vascular resistance and helps maintain hemodynamic perfusion in the skin. However, BP drops as a consequence. (Boulanger, 2016, Ebi et al., 2021) Second, high temperature and excessive humidity cause increased heart rate, which increases BP. (Madaniyazi et al., 2016) Third, recent research shows that vitamin D deficiency is associated with various cardiovascular diseases (including hypertension, cardiac failure, and premature atherosclerosis). (Latic and Vitamin, 2020) Adequate vitamin D exposure when temperature is high inhibits the renin-angiotensin-aldosterone system (RAAS) and reduces oxidative stress, causing lowered BP. In tropical climatic regions during the summer months, there is high production of vitamin D secondary to increased exposure to direct sunlight, which may reduce BP.

The natural indirect electrolyte-mediated effects of ambient temperature on BP can be explained by water and electrolyte loss when the ambient temperature is higher, which may eventually be reflected in urine electrolytes. A normal renal physiological response to increased ambient temperature would be to counteract water and electrolyte losses by activation of the Renin Angiotensin Aldosterone System (RAAS). (Abuelo, 2007) Increased ambient temperature would induce transdermal sodium and water loss, which would lower urine volume and total daily sodium excretion. (Johnson, 1996, Chen et al., 2013) The full spectrum of extracellular volume changes caused by ambient temperature would be counterbalanced by thirst-mediated oral fluid intake (Kenefick, 2018, Noakes, 2007), which is usually adequate to correct perspiration-associated fluid imbalances.

The high salinity of drinking water in the coastal regions of our study area may make this corrective action more effective because people get both water and electrolytes from drinking water. Under perfect physiological regulation, the net effect of water and electrolyte balance would be null, indicating the extra-renal losses would be counterbalanced by the RAAS response and thirst-induced oral fluid intake. The true impact of ambient temperature on BP would thus be dependent on the extent to which individuals can maintain extracellular volume. Several factors like kidney disease, age, use of medications (e.g., diuretics), and unavailability of drinking water can have deleterious effects on extracellular volume regulation. (Abuelo, 2007) Our results suggest that urine electrolytes mediated indirect effect on BP are negligible, suggesting RAAS and thirst mechanisms may have compensated the water and electrolyte loss through sweat in our study population when they were exposed to ambient temperature.

We believe our study has important public health implications in response to changes in ambient temperature due to global climate change. Our findings suggest that mean population BP will be higher when the ambient temperature is lower, and mean population BP will be lower when the ambient temperature increases. The population may benefit from lower BP-related reduced cardiovascular disease (CVD) risk when ambient temperature is higher. Nevertheless, when ambient temperature is extremely high (e.g., heat waves), the BP of vulnerable individuals may fall too much, which may be linked to kidney injuries and other cardiovascular complications. Meta-analyses suggest that the burden of hypertension-related mortalities decreases in higher temperatures and heat waves. (Liu et al., 2022, Kim et al., 2012) Studies suggest that high temperatures and heat waves may increase the burden of cardiovascular disease, (Liu et al., 2022) but such increase burden of cardiovascular disease during heat waves probably cannot be explained by high BP as per our findings and similar findings elsewhere. (Halonen et al., 2011, Kim et al., 2012, Kenney et al., 2014) None of the participants in our study reported the presence of air conditioning in their households. Participants were selected from rural regions of southwest coastal Bangladesh and had low-socio economic conditions. Hence, their indoor temperatures are highly correlated with the ambient temperature without the household air conditioning. Although, we found no effect of ambient heat-induced urine electrolyte-mediated effects on BP, fluid and electrolyte replenishment can help to counterbalance the lowered BP due to the natural direct effect of extremely high heat exposure.

In contrast, when populations are exposed to cold temperatures, CVD risk will be higher due to higher BP. Communities may experience higher BP-related complications (e.g., strokes and heart diseases) during the winter months when ambient temperature is lower. Many studies highlight the U-shaped association between ambient temperature and CVD risk, suggesting a higher CVD burden during extreme cold and heat exposures. (Gostimirovic et al., 2020, Zhai et al., 2023) Our findings suggest that higher BP can contribute to increased CVD risk only during cold exposure. However, a lower BP or other factors may be linked to increased CVD risk during extremely high heat exposure, and thus, further research is needed.

Our study had several strengths. First, we measured every participant’s BP at each visit and used the arithmetic means of three measurements for our analysis. Second, we repeated measures of 24-hour urine biomarkers for five consecutive visits when the BP of the participants was measured. The 24-hour electrolyte is a better proxy of daily electrolyte balance than the spot urine electrolytes or equation-estimated urine electrolytes. (Naser et al., 2021) Therefore, we had fewer measurement errors in urine electrolyte mediators due to reliance on 24-hour urine sample collection. Several large-scale epidemiological studies recommend repeated measurements to account for individual-level variations. (Philippat and Calafat, 2021, Singh et al., 2013) Moreover, ambient temperature-induced fluid and electrolyte loss through sweat and BP changes occur within minutes to hours. In this study, we linked the same-day ambient temperature, urine electrolytes and volume, and blood pressure of the participants for five visits, allowing us a better understanding of ambient temperature-induced effects on BP. Finally, random intercepts for individual-level statistical analysis controls for unmeasured individual-level confounders.

Our study had several limitations. First, we used spatiotemporally linked temperature data, which may lead to exposure measurement error. (Colston et al., 2018) We collected the temperature data from BMD, which has more than thirty climate stations throughout the country. Even though weather-station-based meteorological measurements are thought to be representative of actual local conditions, households located at far distances may have different meteorological conditions. (Mistry et al., 2022) This can thus cause inaccuracies in exposure measurement that may lead to exposure measurement error. Nevertheless, the temperature among the three weather stations had a very high correlation. Collecting individual household level data on temperature is costly, and thus, temperature data from weather stations are often used for research in Bangladesh.

Second, we did not collect sweat samples to measure the water and electrolyte loss through sweat and for water and electrolyte intake in response to ambient heat. We assumed that changes in water and electrolyte balance would be reflected in urine volume and electrolytes’ excretion. Nevertheless, urine electrolyte levels may not be an ideal proxy of water and electrolyte balance due to the presence of several additional physiological counter-regulatory mechanisms, including the RAAS and other neurohumoral systems that regulate water and electrolyte balance. Finally, since southwest coastal Bangladesh is located in the tropical region and we conducted the study during winter and early summer, we were unable to study the effects of low ambient temperature. Similarly, we did not have data for late summer. There are no significant temperature differences between early and late summer in Bangladesh. (Weather Atlas, 2024) Therefore, our study findings may have limited generalizability in other climatic conditions except for tropic regions.

## 5 Conclusions

Our study suggests exposure to ambient temperature can inversely affect population BP; therefore, ambient temperature needs to be included in the clinical management of high BP and related complications. Hypertension-related adverse health outcomes are likely to occur when ambient temperature is cold, and hypohydration-related complications are likely to occur when temperature is higher. Since the negative effect of ambient temperature is significant, our findings highlight the importance of maintaining the temperature of residences and workplaces when populations across the world are increasingly experiencing higher ambient heat.

We also need further research to better understand the pathophysiological role of ambient temperature variations on BP, hypertension control, and cardiovascular disease burden by collecting precise individual-level data on heat exposure, water and electrolyte balance, RAAS, and antidiuretic hormone (ADH) response, along with continuous measurement of BP for better population-level intervention and clinical management of BP in response to global climate change.

## Conflict of interest

Dr. Kovesdy received honoraria for consultant work unrelated to the present study from Abbott, Akebia, Astra Zeneca, Bayer, Boehringer Ingelheim, Cara Therapeutics, CSL Behring, CSL Vifor, GSK, Pharmacosmos, Rockwell and Takeda.

## CRediT authorship contribution statement

**Ayesha Mukhopadhyay:** Writing – review & editing, Writing – original draft, Methodology, Formal analysis. **Momenul Haque Mondol:** Writing – review & editing, Visualization, Validation, Methodology, Formal analysis. **Mahbubur Rahman:** Writing – review & editing, Resources, Methodology, Investigation, Funding acquisition. **Leanne Unicomb:** Writing – review & editing, Resources, Methodology, Funding acquisition. **Rizwana Khan:** Writing – review & editing, Methodology. **Hoimonty Mazumder:** Writing – review & editing, Formal analysis. **Mohammad Nahian Ferdous:** Writing – review & editing, Formal analysis. **Emily V. Pickering:** Writing – review & editing, Resources. **Konstantinos C. Makris:** Writing – review & editing, Resources, Formal analysis. **Alberto J. Caban-Martinez:** Writing – review & editing, Supervision, Resources. **Faruk Ahmed:** Writing – review & editing, Resources, Methodology. **Mohammad Shamsudduha:** Writing – review & editing, Formal analysis. **Fawaz Mzayek:** Writing – review & editing, Resources. **Chunrong Jia:** Writing – review & editing, Supervision, Methodology. **Hongmei Zhang:** Writing – review & editing, Supervision, Resources, Formal analysis, Data curation. **Anwar Musah:** Writing – review & editing, Supervision, Resources. **Lora E. Fleming:** Writing – review & editing, Supervision, Resources. **Matthew P. Smeltzer:** Writing – review & editing, Supervision, Resources. **Howard H. Chang:** Writing – review & editing, Supervision, Resources, Formal analysis, Data curation. **John L. Jefferies:** Writing – review & editing, Resources. **Csaba P. Kovesdy:** Writing – review & editing, Supervision, Resources, Methodology. **Xichen Mou:** Writing – review & editing, Supervision, Software, Methodology, Formal analysis, Data curation. **Abu Mohd Naser:** Writing – review & editing, Visualization, Methodology, Investigation, Funding acquisition, Formal analysis, Conceptualization.

## Funding

The study has been funded by 10.13039/100010269Wellcome Trust (UK) through an award under the *Our Planet, Our Health* Programme (Grant# 106871/Z/15/Z). Dr. Abu Mohd Naser's time was partly supported by an 10.13039/100000002NIH grant (grant# R15ES035227-01).

## Declaration of competing interest

The authors declare that they have no known competing financial interests or personal relationships that could have appeared to influence the work reported in this paper.

## Data Availability

Data will be made available on request.
