# Supplementary material for: The direct and urinary electrolyte-mediated effects of ambient temperature on population blood pressure: A causal mediation analysis
Source: Environ Int. 2025 Jan;195:109208. doi: 10.1016/j.envint.2024.109208 (PMC11757155; doi:10.1016/j.envint.2024.109208)
Supplement: Supplementary Data 1 [file mmc1.docx]

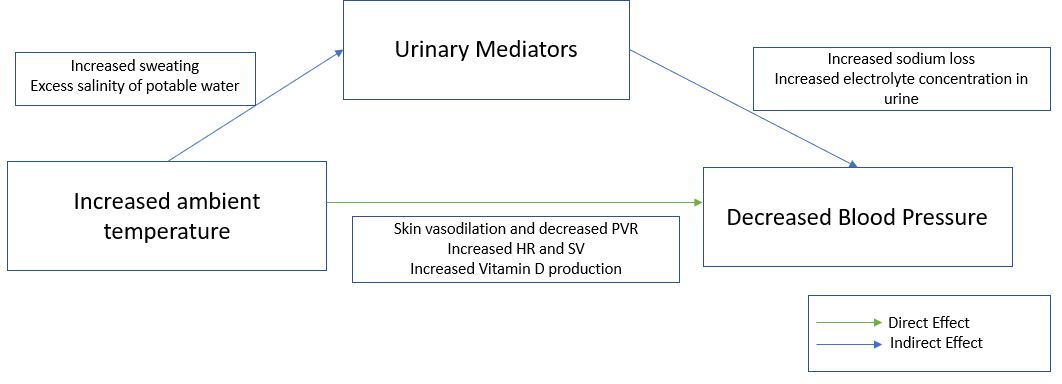


Supplemental Figure 1: Directed acyclic graph. Abbreviations: PVR, Peripheral Vascular Resistance; HR, Heart Rate; SV, Stroke Volume.


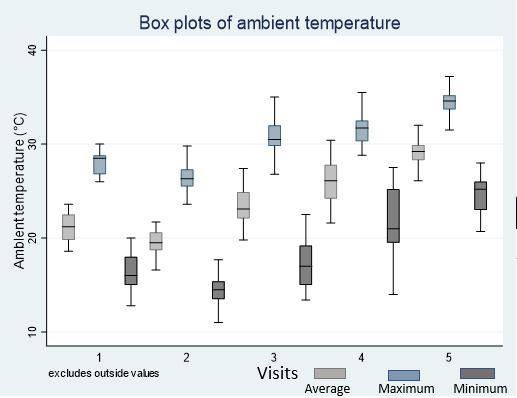


Supplemental Figure 2a: Box plots for relationship between visits and ambient temperature


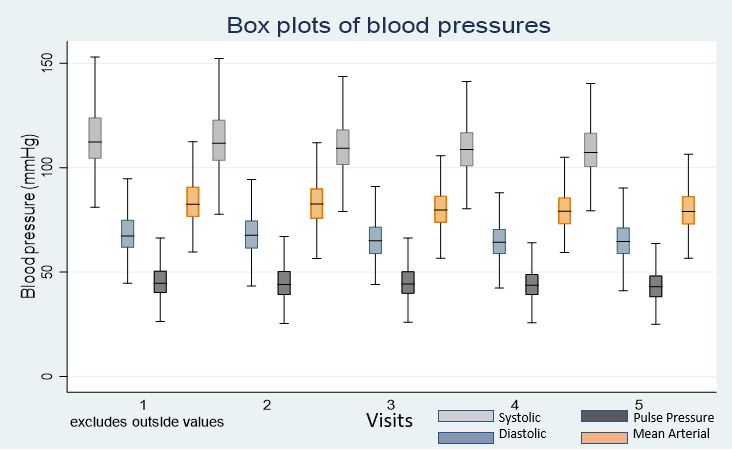


*Supplemental Figure 2b: Box plots for relationship between visits and blood pressures*

Supplemental Figure 3: Pearson correlation coefficient between daily average temperature in three weather stations located in the study area. “s_avgtemp” indicates daily average temperature of Satkhira weather station; “k_avgtemp” indicates daily average temperature of Khulna weather station, and “m_avgtemp” indicates daily average temperature of Bagerhat weather station.

Supplemental Table 1: Relationship between 5°C increase in average daily temperature and blood pressure when multiple mediators were used in the same model after excluding participants who reported the used of antihypertensive medications. “NDE” effect indicates natural direct effect, and “NIE” indicates natural indirect effect. Abbreviations: Na, Sodium; K, Potassium; Ca, Calcium; Mg, Magnesium; 95% CI, 95% confidence interval; BP, blood pressure; sbp, systolic blood pressure; and dbp, diastolic blood pressure.

| Effect type | BP types | effect | 95% confidence interval | |
| --- | --- | --- | --- | --- |
|  |  |  | lower limit | upper limit |
| Na (NIE) | SBP | -0.0240 | -0.0487 | -0.0061 |
| K (NIE) | SBP | 0.0377 | 0.0194 | 0.0581 |
| Ca (NIE) | SBP | 0.0176 | 0.0024 | 0.0347 |
| Mg (NIE) | SBP | -0.0005 | -0.0097 | 0.0063 |
| Summation of Na.K.Ca.Mg (NIE) | SBP | 0.0305 | 0.0082 | 0.0526 |
| Direct (NDE) | SBP | -0.3160 | -0.4631 | -0.1975 |
| Total effect | SBP | -0.2855 | -0.4296 | -0.1774 |
| Na (NIE) | DBP | -0.0052 | -0.0153 | 0.0005 |
| K (NIE) | DBP | 0.0151 | 0.0061 | 0.0224 |
| Ca (NIE) | DBP | 0.0262 | 0.0138 | 0.0397 |
| Mg (NIE) | DBP | 0.0086 | 0.0018 | 0.0183 |
| Summation of Na.K.Ca.Mg (NIE) | DBP | 0.0447 | 0.0251 | 0.0585 |
| Direct (NDE) | DBP | -0.2406 | -0.3123 | -0.1600 |
| Total effect | DBP | -0.1959 | -0.2696 | -0.1135 |

Supplemental Table 2: Relationship between 5°C increase in average daily temperature and blood pressure when multiple mediators were used in the same model among participants who had complete 24-hour samples based on creatinine index >0.7. “NDE” effect indicates natural direct effect, and “NIE” indicates natural indirect effect. Abbreviations: Na, Sodium; K, Potassium; Ca, Calcium; Mg, Magnesium; 95% CI, 95% confidence interval; BP, blood pressure; sbp, systolic blood pressure; and dbp, diastolic blood pressure.

| Effect type | BP types | effect | 95% confidence interval | |
| --- | --- | --- | --- | --- |
|  |  |  | lower limit | upper limit |
| Na (NIE) | SBP | -0.0260 | -0.0450 | -0.0099 |
| K (NIE) | SBP | 0.0404 | 0.0253 | 0.0611 |
| Ca (NIE) | SBP | 0.0198 | 0.0042 | 0.0403 |
| Mg (NIE) | SBP | 0.0033 | -0.0063 | 0.0148 |
| Summation of Na.K.Ca.Mg (NIE) | SBP | 0.0376 | 0.0133 | 0.0651 |
| Direct (NDE) | SBP | -0.3372 | -0.4755 | -0.2053 |
| Total effect | SBP | -0.2997 | -0.4338 | -0.1561 |
| Na (NIE) | DBP | -0.0045 | -0.0143 | 0.0017 |
| K (NIE) | DBP | 0.0144 | 0.0043 | 0.0278 |
| Ca (NIE) | DBP | 0.0245 | 0.0117 | 0.0397 |
| Mg (NIE) | DBP | 0.0084 | 0.0008 | 0.0181 |
| Summation of Na.K.Ca.Mg (NIE) | DBP | 0.0428 | 0.0245 | 0.0638 |
| Direct (NDE) | DBP | -0.2488 | -0.3192 | -0.1758 |
| Total effect | DBP | -0.2060 | -0.2726 | -0.1283 |

*Supplemental Table 3: P-values for the interaction terms between temperature and mediator for the relationship between 5°C increase in daily temperature and blood pressure.*

| **Exposure** | **Mediator** | **outcome** | **p-value* for exposure-mediator interaction term using Wald test** |
| --- | --- | --- | --- |
| Average ambient temperature | 24-hour urine Na | SBP | 0.747 |
| Average ambient temperature | 24-hour urine Na | DBP | 0.614 |
| Average ambient temperature | 24-hour urine K | SBP | 0.675 |
| Average ambient temperature | 24-hour urine K | DBP | 0.629 |
|  |  |  |  |
| Maximum ambient temperature | 24-hour urine Na | SBP | 0.698 |
| Maximum ambient temperature | 24-hour urine Na | DBP | 0.923 |
| Maximum ambient temperature | 24-hour urine K | SBP | 0.977 |
| Maximum ambient temperature | 24-hour urine K | DBP | 0.445 |
|  |  |  |  |
| Minimum ambient temperature | 24-hour urine Na | SBP | 0.536 |
| Minimum ambient temperature | 24-hour urine Na | DBP | 0.331 |
| Minimum ambient temperature | 24-hour urine K | SBP | 0.404 |
| Minimum ambient temperature | 24-hour urine K | DBP | 0.931 |

“SBP” indicates systolic blood pressure, and “DBP” indicates diastolic blood pressure. * p-values were determined by Wald test.
